# Treatment response after chemoradiation with an external beam radiation therapy boost in cervical cancer patients in Rwanda

**DOI:** 10.1093/oncolo/oyaf328

**Published:** 2025-10-01

**Authors:** Dieudonne Duhoranenayo, Sabine Esperance Nyiraneza, Eulade Rugengamanzi, Richard Sungura, Emmanuel Rudakemwa, Cherise Umutoni Gahizi, Joseph Musabyimana, Theoneste Maniragaba, Pacifique Nikuze, Jean Jacques Nshizirungu, Jean Paul Ruboneka, Rafi Kabarriti, Fidel Rubagumya

**Affiliations:** Imaging Department, University Teaching Hospital of Kigali (CHUK) (Centre Hospitalier Universitaire de Kigali), Kigali 655, Rwanda; Imaging Department, University Teaching Hospital of Kigali (CHUK) (Centre Hospitalier Universitaire de Kigali), Kigali 655, Rwanda; Diagnostic Imaging and Radiation Sciences Department, College of Medicine and Health Sciences, University of Rwanda, Kigali 4285, Rwanda; Oncology Department, Butaro Hospital, Burera, Musanze 59, Rwanda; Imaging Department, University Teaching Hospital of Kigali (CHUK) (Centre Hospitalier Universitaire de Kigali), Kigali 655, Rwanda; Medical Imaging Department, Rwanda Military Hospital (RMH), Kigali 3377, Rwanda; Imaging and Diagnostic Department, King Faisal Hospital, Kigali 2534, Rwanda; Imaging Department, University Teaching Hospital of Kigali (CHUK) (Centre Hospitalier Universitaire de Kigali), Kigali 655, Rwanda; Oncology Department, Rwanda Military Hospital (RMH), Kigali 3377, Rwanda; Cancer Disease Program, Non-Communicable Diseases Division, Rwanda Biomedical Centre, Kigali 7162, Rwanda; Preventive and Community Dentistry Department, College of Medicine and Health Sciences, University of Rwanda, Kigali 4285, Rwanda; Imaging and Diagnostic Department, King Faisal Hospital, Kigali 2534, Rwanda; Imaging and Diagnostic Department, King Faisal Hospital, Kigali 2534, Rwanda; Albert Einstein College of Medicine, New York, NY 10461, United States; Oncology Department, Rwanda Military Hospital (RMH), Kigali 3377, Rwanda; Medical Oncology, College of Medicine and Health Sciences, University of Rwanda, Kigali 4285, Rwanda

**Keywords:** cervical cancer, chemoradiation therapy, MRI, treatment response and RECIST criteria

## Abstract

**Background:**

Cervical cancer remains a significant public health burden in Rwanda, where brachytherapy is not widely available. This study assessed treatment response in cervical cancer patients post-chemoradiation using magnetic resonance imaging (MRI)-based Response Evaluation Criteria in Solid Tumors (RECIST), focusing on the effectiveness of external beam boost as an alternative.

**Patients and Methods:**

This retrospective study was conducted at the Rwanda Cancer Centre, including patients treated with chemoradiation followed by an external beam boost from January 2020 to June 2022. MRI scans performed before treatment and 3-6 months post-treatment were analyzed using RECIST criteria to classify treatment response as complete response (CR), partial response (PR), stable disease (SD), or progressive disease (PD). Agreement between clinical and MRI staging pre- and post-treatment was assessed using kappa coefficients.

**Results:**

Eighty-eight patients were included (mean age: 57.7 ± 10.6 years). CR, PR, SD, and PD were observed in 67%, 17%, 9%, and 7% of patients, respectively. MRI findings demonstrated substantial agreement with clinical staging before pre-treatment (78%, *K* = 0.63) and after post-treatment (71%, *K* = 0.71). CR rates were highest in early-stage disease (FIGO stage I: 90%), whereas PD was more frequent in advanced stages (FIGO stage II: 9%; FIGO stage III: 13%).

**Conclusion:**

The MRI-based RECIST criteria effectively assess the cervical cancer treatment response after post-chemoradiation. The high CR rate (67%) suggests that an external beam boost may serve as a viable alternative for brachytherapy. However, PD in advanced-stage disease highlights the need for further research to optimize treatment strategies. Future studies should evaluate long-term outcomes and explore advanced MRI techniques to enhance the response assessment.

Implications for Practice This study demonstrates that MRI-based Response Evaluation Criteria in Solid Tumors effectively evaluate treatment response in cervical cancer patients receiving chemoradiation with an external beam boost, offering a practical alternative in settings where brachytherapy is unavailable. The high complete response rate (67%) supports the clinical utility of this approach, particularly in early-stage disease. However, the persistence of progressive disease in advanced stages underscores the need for optimized strategies. These findings are crucial for low-resource regions, providing evidence-based guidance for treatment monitoring and reinforcing MRI’s role in improving cervical cancer management. Future research should explore long-term outcomes and advanced imaging to refine response assessment.

## Introduction

Cervical cancer is the fourth most prevalent cancer in females worldwide.^1^ In 2022, 348,874 females were newly diagnosed of cervical cancer worldwide, and approximately 662,301 were diagnosed with the disease in the same year.^1^ Cervical cancer affects 22.5% of females in Sub-Saharan Africa who are found to have cancer and majority of these women reside in rural regions.^2^ One of the most affected areas is East Africa, where there are more than 30 cases per hundred thousand women each year.^3^ The most alarming uniform increases have recently been identified in 7 out of 8 nations in sub-Saharan: Seychelles Kenya, Malawi, Gambia, South Africa, Uganda, and Zimbabwe.^4^ In Rwanda, the most common type of cancer in women is cervical cancer, with 866 new cases, and approximately 609 patients die of this cancer according to the Globocan data 2022.^1^ Within the population of 11 million, 2.72 million Rwandan women above the age of 15 years are at risk of developing cervical cancer^5^; however, this number is expected to significantly decrease because of the nationwide Human Papilloma Virus vaccination program.^6^

The standard treatment for locally advanced cervical cancer typically involves external beam radiation therapy (EBRT) combined with concurrent chemotherapy followed by brachytherapy boost. Brachytherapy has been shown to improve local control and overall survival rates.^7^ However, in Rwanda, because of the limited availability of brachytherapy, an external beam boost is often used as an alternative. This substitution raises questions about the comparative efficacy of EBRT with an external beam boost versus the standard brachytherapy boost, particularly concerning the treatment response rates.

Accurate assessment of treatment response is crucial in guiding further management, decision-making, and prediction of patient outcomes.^8^ Methods used to assess treatment response, such as clinical examination and imaging with computed tomography (CT) or magnetic resonance imaging (MRI), have limitations in accurately detecting residual disease or distinguishing between fibrosis and viable tumor tissue post-chemoradiation.^9^ However, MRI offers advantages over other imaging modalities for evaluating treatment response in patients with cervical cancer.^10^ It provides excellent soft tissue contrast, multiplanar imaging capabilities, and the ability to perform functional imaging techniques such as diffusion-weighted imaging and dynamic contrast-enhanced MRI (DCE-MRI). These techniques can assess tumor viability, perfusion, and microstructural changes, which are valuable for detecting residual disease and predicting treatment outcomes.^11^ The Response Evaluation Criteria in Solid Tumors (RECIST) is commonly used to assess treatment response in solid tumors.^12^

Despite the potential of MRI in assessing treatment response, MRI remains an inaccessible (both in terms of availability and affordability) diagnostic imaging modality in lower middle-income countries. At the time of this study, there were 2 MRI machines distributed in 2 private hospitals. Additionally, standardized protocols and criteria for interpreting MRI findings in patients with cervical cancer post-chemoradiation are lacking.^13^

For patients treated for locally advanced cervical cancer and able to perform both pre- and post-treatment pelvic MRI, this study aimed to assess their treatment response using MRI-based RECIST criteria.

## Methods

### Study design and settings

This was a retrospective study conducted from January 1, 2020 to June 30, 2022. This timeframe was chosen because radiotherapy services at the Rwanda Cancer Center started in January 2020, marking the beginning of standardized treatment for cervical cancer patients in the country. Additionally, June 2022 was selected as the endpoint to ensure the availability of post-chemoradiation imaging conducted within the 3-6 months follow-up period, allowing for a comprehensive assessment of treatment response using the MRI-based RECIST criteria.

RECIST were used in image evaluation to assess the treatment response. The RCC is located inside the Rwanda Military Referral and Teaching Hospital (RMRTH) was the study site. RMRTH is a referral and teaching hospital situated in the Kicukiro District, Kigali City, with a bed capacity of 414. The RCC located within RMRTH started offering treatment in March 2019 with 2 linear accelerator machines using Volumetric Modulated Arc Therapy (VMAT), 1 CT simulator for treatment planning.

### Study population

The study included all patients with histologically confirmed cervical cancer treated with concurrent chemoradiation and pelvic MRI images before and after chemoradiation. Only the first post-chemoradiation pelvic MRI, performed between 6 and 10 weeks, was considered. Patients treated for cervical cancer recurrence were excluded from the study.

### Treatment response assessment

RECIST were used to assess treatment response in solid tumors.^12^ The total disappearance of all target lesions was considered radiological complete response (CR), and any pathological lymph node, target or non-target, had to have the short axis reduced to less than 10 mm. Partial response (PR) was defined as a decrease of at least 30% in the total diameter of target lesions. A 20% increase in the total diameters of the target lesions, using the smallest sum as a reference (including the baseline sum if it was the smallest), was considered a progressive disease (PD). Furthermore, relative growth in new or additional lesions was considered progression. The definition of the stable disease (SD) was defined as insufficient shrinkage to be eligible for PR or PD, referring to the smallest sum diameters.^14^

### Analysis

Stata 14 was used for data processing and visualization. While averages and standard deviations were considered to show quantitative data, percentages were used to describe the qualitative aspects of treatment response. The kappa coefficient and percent agreement were used to show the link between clinical staging and MRI findings; a *P*-value less than 5% was deemed statistically significant.

## Results

**Figure 1.**
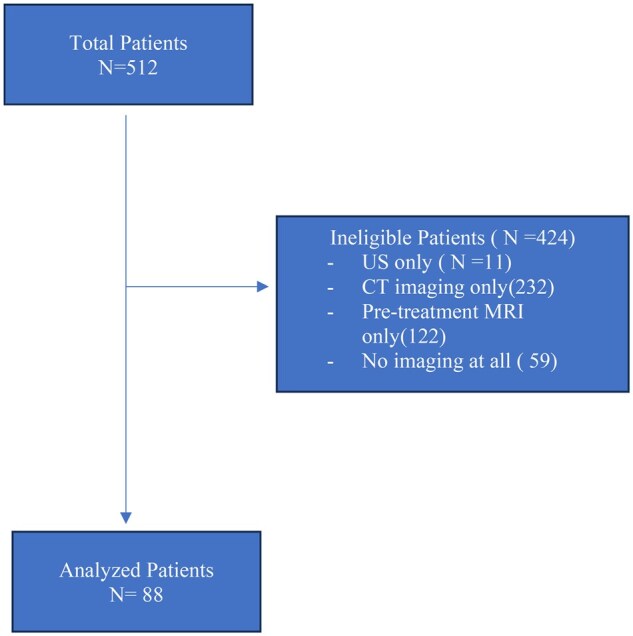
A Consort diagram showing patients selection.

At the time of data collection, 512 patients with clinically and histologically confirmed cervical cancer were seen at the RCC and were referred from different health facilities. Each patient was examined individually, and the required data were recorded. Ultimately, 424 patients were ineligible for analysis because different exclusion criteria were met. A total of 88 patients were eligible for the final analysis (Figure 1).

### Patient characteristics

The baseline characteristics of the study population are summarized in Table 1. The mean age of the patients was 57.7 years (±10.6) years, with the majority (79%) being over 50 years old. Performance status, as assessed using the Eastern Cooperative Oncology Group (ECOG) scale, was predominantly ECOG 0 (41%) and ECOG 1 (48%), with a small proportion of patients with ECOG 2 (8%) or ECOG 3 (2.3%). Histopathological evaluation revealed that squamous cell carcinoma was the most common subtype, accounting for 97% of cases, whereas adenocarcinoma was present in 3% of patients. The majority of tumors were grade II (57%), followed by grade I (13%) and grade III (8%). Pretreatment clinical staging was classified as stage IB in 11% of the patients, stage IIA in 20%, stage IIB in 28%, stage IIIA in 17%, stage IIIB in 18%, and stage IV in 5%. Nodal involvement was observed in 25% of the patients. Pretreatment MRI staging showed a similar distribution, with the majority of patients staged as stage IIA (25.0%) and IIB (20%).

**Table 1.** Age, performance status, histopathology evaluation, and staging.

Variables	Categories	Frequency	%
**Age (years)**			
**Mean (standard deviation)**	57.7(±10.6)		
**Age group**			
	<50	19	21
	>50	69	79
**ECOG**			
	0	37	41
	1	42	48
	2	7	8
	3	2	2
**Histology grade**			
	Grade I	11	13
	Grade II	50	57
	Grade III	7	8
	Grade IV	1	1
	Unknown	19	22
**Histopathological subtypes**			
	SCC	85	97
	Adenocarcinoma	3	3
**Pretreatment clinical staging**			
	IB	10	11
	IIA	18	20
	IIB	25	28
	IIIA	15	17
	IIIB	16	18
	IVA	4	5
**Nodal involvement**	Yes	22	25
	No	66	75
**Pretreatment MRI staging**			
	IB	8	9
	IIA	22	25
	IIB	18	21
	IIIA	14	16
	IIIB	13	15
	IIIC	2	2
	IVA	10	11
	IVB	1	1

### Concordance between pretreatment clinical staging and MRI findings

The concordance between pre-treatment clinical (pelvic physical examination) staging and MRI staging in patients with cervical cancer is presented in Table 2. Imaging was not used during clinical staging. Among the 88 patients evaluated, the highest concordance was observed in stage IIB, with 14 of 20 cases (70%) correctly staged by MRI. Stage IIA also showed substantial agreement, with 16 of 18 cases (89%) being accurately staged. However, discrepancies were noted in stages IIIA and IIIB, where MRI findings identified additional cases of parametrial and pelvic sidewall invasion that were not detected by clinical staging alone. Overall, the total concordance rate across all stages was 78%, with a κ statistic of 0.63 (*P* < .001), indicating good agreement between the clinical staging and MRI findings (Table 3).

**Table 2.** Concordance between pretreatment clinical staging and MRI staging.

				MRI					
Clinical Staging	IB	IIA	IIB	IIIA	IIIB	IIIC	IVA	IVB	Total
**IB**	7	2	1	-	-	-	-	-	10 (11)
**IIA**	1	16	1	-	-	-	-	-	18 (20)
**IIB**	-	2	14	2	1	-	1	-	20 (23)
**IIIA**	-	-	1	9	-	-	3	1	14 (16)
**IIIB**	-	2	1	3	10	1	4	-	21 (24)
**IVA**	-	-	-	-	2	1	2	-	5 (6)
**IVB**	-	-	-	-	-	-	-	-	0 (0)
**Total**	8 (9)	22 (25)	18 (20)	14 (16)	13 (15)	2 (2)	10 (11)	1 (1)	88

**Table 3.** Pre-treatment agreement between clinical staging and pelvis MRI staging in cervical patients.

Site	Agreement (%)	κ	*P*
**Parametrial invasion**	82%	0.69	.001
**Upper vaginal 2/3 invasion**	70%	0.26	.023
**Lower 1/3 invasion**	75%	0.52	.046
**Pelvic side wall invasion**	72%	0.44	.001
**Overall**	78%	0.63	.001

### Pretreatment agreement between clinical staging and pelvis MRI findings

Table 3 details the pre-treatment agreement between clinical staging and pelvis MRI findings for specific sites of invasion. The highest agreement was observed for parametrial invasion (82%, κ = 0.69, *P* < .001), followed by pelvic sidewall invasion (72%, κ = 0.44, *P* < .001). Lower agreement was noted for upper vaginal 2/3 invasion (70%, κ = 0.26, *P* = .023) and lower 1/3 vaginal invasion (75%, κ = 0.52, *P* = .046). The overall agreement across all sites was 78% (κ = 0.63, *P* < .001).

### Post-treatment agreement between clinical and pelvis MRI findings

The post-treatment agreement between the clinical and MRI findings is summarized in Table 4. Only the first post-chemoradiation pelvic MRI was reviewed, with a median interval of 8 weeks after the completion of chemoradiation. The overall agreement was 71% (κ = 0.71, *P* < .001), with the lowest agreement observed for the lower 1/3 vaginal invasion (76%, κ = 0.62, *P* < .001) and pelvic sidewall invasion (77%, κ = 0.53, *P* < .001). Parametrial invasion and upper vaginal 2/3 invasion also showed good agreement, with rates of 80% (κ = 0.61, *P* < .001) and 78% (κ = 0.52, *P* < .001), respectively.

**Table 4.** Post-treatment agreement between clinical staging and pelvis MRI staging in cervical cancer patients.

Site	Agreement (%)	κ	*P*
**Parametrial invasion**	80%	0.61	.001
**Vaginal 2/3 invasion**	78%	0.52	.001
**Lower 1/3 Invasion**	76%	0.62	.001
**Pelvic side wall Invasion**	77%	0.53	.001
**Overall**	71%	0.71	.001

### Treatment response based on RECIST criteria

Table 5 outlines the treatment responses based on the RECIST criteria following chemoradiation. CR was achieved in 59 patients (67%), while 15 patients (17%) exhibited PR. PD and SD were observed in 8 (9%) and 6 (7%) patients, respectively.

**Table 5.** Treatment response based on RECIST criteria after chemoradiation.

Treatment response	Number	%
**Complete response**	59	67
**Partial response**	15	17
**Progressive disease**	8	9
**Stable disease**	6	7

### Distribution of treatment responses across FIGO stages

The distribution of treatment responses according to the RECIST criteria across the different FIGO stages is presented in Table 6. Patients with FIGO stage I had the highest rate of CR (90%), followed by those with FIGO stage II (70%) and FIGO stage III (65%). No CRs were observed in FIGO stage IV patients, with all cases showing either stable or PD. The differences in treatment responses across the stages were statistically significant (*P* < .001).

**Table 6.** Distribution of treatment responses according to RECIST criteria across different Figo stages.

Stages	CR (%)	PR (%)	PD (%)	SD (%)	*P* value
**Figo I**	9 (90)	1 (10)	0 (0)	0 (0)	.001
**Figo II**	30 (70)	9 (21)	4 (9)	0 (0)	
**Figo III**	20 (65)	5 (16)	4 (13)	2 (6)	
**Figo IV**	0 (0)	0 (0)	0 (0)	4 (100)	

## Discussion

This study assessed treatment response in cervical cancer patients post-chemoradiation in Rwanda, using MRI-based RECIST criteria. Because of the limited availability of brachytherapy, an external beam boost is utilized as an alternative. Five major findings emerged: First, the majority of patients showed CR. Second, there was strong concordance between the clinical and MRI findings, with a substantial pre-treatment agreement. Third, the post-treatment MRI findings continued to show good agreement with the clinical staging across most invasion sites.

Fourth, MRI provided enhanced detection of parametrial and pelvic sidewall invasion that was not apparent through clinical staging alone.

Fifth, treatment response varied significantly according to FIGO stage, with higher CR rates observed in early-stage disease.

Recent studies have explored the efficacy of EBRT in cervical cancer treatment, particularly when brachytherapy is not feasible. For instance, a multicenter retrospective study by the Korean Radiation Oncology Group reported that approximately 61% of patients showed CR following EBRT boost, with a 5-year local failure-free survival rate of 70%. Similarly, a study from the National Institute of Oncology in Rabat observed an 80.5% CR rate among patients receiving EBRT boost, with 5-year overall survival and disease-free survival rates of 47% and 44%, respectively.^15^

In comparison, our study demonstrated a 67% CR rate with EBRT boost, aligning with these findings and suggesting that EBRT boost can be a viable alternative in settings where brachytherapy is unavailable. However, it is important to note that brachytherapy has been associated with higher CR rates. For example, a study comparing the outcomes of cervical ­cancer patients treated with and without brachytherapy found a 92.5% CR rate in the brachytherapy group versus 73.3% in the EBRT-alone group. Another study reported a 95.7% CR rate with short-course brachytherapy.^16^^,^^17^ In addition, our findings on CR align with findings from previous studies, where whole pelvic radiation followed by a brachytherapy boost resulted in CR rates ranging from 60% to 80%.^18^ However, this study differs in that an external beam boost was used instead of brachytherapy, which has been reported to yield slightly lower CR rates.^19^

The relatively high CR rate (67%) in our study, despite the lack of brachytherapy, is encouraging. This suggests that an external beam boost may serve as a viable alternative in settings where brachytherapy is unavailable. One possible reason for this outcome is the use of advanced radiation techniques, such as VMAT, which optimizes dose delivery and spares surrounding organs. However, studies have indicated that brachytherapy offers superior dose conformity.^20^ Thus, while external beam boost shows promise, it may still be less effective in achieving local tumor control than brachytherapy.

The PR rate of 17% in our study is consistent with the literature, as studies suggest that approximately 15%-25% of patients may not achieve complete tumor regression post-chemoradiation.^21^ The rate of PD (9%) in our study was slightly lower than the global estimates (10%-15%),^20^ which may be attributed to differences in patient selection, treatment protocols, or follow-up duration.

Our study revealed that disease progression was more common in patients with advanced-stage disease, particularly in FIGO stages II and III, with 9% and 13% patients presenting PD, respectively. This is consistent with prior research, which indicated that patients with larger tumor burdens and nodal involvement have a higher risk of treatment failure.^22^ One potential explanation is that tumors in advanced stages may have hypoxic regions, reducing radiosensitivity and increasing the likelihood of residual disease post-treatment. This underscores the need for intensified treatment strategies such as dose escalation or alternative therapies for high-risk patients.

The strong agreement between MRI findings and clinical staging supports the existing evidence that MRI is a superior imaging modality for tumor response assessment compared with clinical examination alone.^13^ Radiological imaging plays a pivotal role in the accurate staging and management of cervical cancer, often providing more precise information than clinical assessment alone. In our study, the concordance between clinical staging and MRI findings was high (78.1%, κ = 0.63), underscoring MRI's superior ability of MRI to assess the local tumor extent, parametrial invasion, and pelvic sidewall involvement. This is consistent with multiple studies demonstrating that MRI has the highest accuracy among imaging modalities for evaluating local tumor size and extent, outperforming clinical examination, ultrasound, and CT for soft tissue delineation.^23^ Despite this, the FIGO staging system, revised in 2018, still primarily recommends clinical examination, chest X-ray, ultrasound, and optional MRI or CT if available—reflecting a pragmatic approach for global applicability, especially in low-resource settings. However, this reliance on clinical staging can lead to underestimation or overestimation of the disease, particularly for parametrial and nodal involvement. PET/CT has shown great utility in detecting metastatic or nodal disease, with higher sensitivity than MRI or CT alone, and is often recommended for treatment planning in high-resource settings.^24^ Nevertheless, its limited availability and high cost restrict its routine use in many low-income and middle-income countries. Our findings support the growing body of evidence that MRI should be integrated more formally into the staging algorithm, particularly in accessible centers, to improve staging accuracy and guide appropriate treatment strategies. These findings highlight the need for policy interventions to improve the outcomes of cervical cancer treatment in Rwanda. Given the demonstrated efficacy of external beam boost, policymakers should consider optimizing EBRT protocols in settings in which brachytherapy is unavailable. However, efforts should also be directed toward expanding the access to brachytherapy, as it remains the gold standard for cervical cancer treatment. Additionally, the study underscores the importance of MRI in response assessment, advocating its routine integration into treatment evaluation protocols. Establishing national guidelines for MRI-based response assessments could enhance treatment monitoring and decision making. Furthermore, this study supports the expansion of cervical cancer screening programs to detect the disease at earlier stages, where treatment response is more favorable.

This study had several limitations. First, its retrospective design may introduce selection bias, as only patients with pre- and post-treatment MRI were included. This may exclude a subset of patients who were clinically evaluated but did not undergo pre-or post-treatment MRI. Second, the relatively small sample size (88 patients) limited the generalizability of the findings. Third, the lack of long-term follow-up data restricts the ability to assess recurrence rates and overall survival. Despite these limitations, this study had several strengths. To our knowledge, this is the first study in Rwanda and Sub-Sahara Africa to evaluate cervical cancer treatment response using MRI-based RECIST criteria in cervical cancer patients treated with EBRT boost instead of brachytherapy boost. The study also benefited from the use of standardized imaging criteria, which enhanced the reliability of the response assessment.

## Conclusion

This study provides critical insights into the treatment response of patients with cervical cancer undergoing chemoradiation with an external beam boost in Rwanda. The high CR rate suggests that an external beam boost may be a viable alternative in resource-limited settings, although efforts to increase access to brachytherapy should continue. The observed disease progression in the advanced stages highlights the need for treatment intensification in high-risk patients. These findings underscore the importance of optimizing radiotherapy protocols, expanding imaging capabilities, and strengthening early detection programs to improve cervical cancer outcomes in Rwanda.

## Data Availability

The data that support the findings of this study are available on request from the corresponding author. The data are not publicly available due to their containing information that could compromise the privacy of research participants.
